# Supplementary material for: Multiple treatment interruptions and protecting HIV-specific CD4 T cells enable durable CD8 T cell response and viral control
Source: Front Med (Lausanne). 2024 May 14;11:1342476. doi: 10.3389/fmed.2024.1342476 (PMC11130509; doi:10.3389/fmed.2024.1342476)
Supplement: Supplementary file 1 [file Data_Sheet_1.PDF]

Multiple Treatment Interruptions and Protecting HIV-Specific CD4 T-Cells Enables Durable CTL Response and Viral Control

Anshika Jain[1], Gaspar E. Canepa[1], Mei-Ling Liou[1], Emily L. Fledderman[1], Andrei I. Chapoval[1], Lingzhi Xiao[1], Ipsita Mukherjee[1], Bushirat M. Balogun[1], Hellen Huaman-Vergara[1], Jeffrey A. Galvin[1], Princy N. Kumar[2], José Bordon[3], Marcus A. Conant[1], Jefferey S. Boyle[1]

Supplementary Data

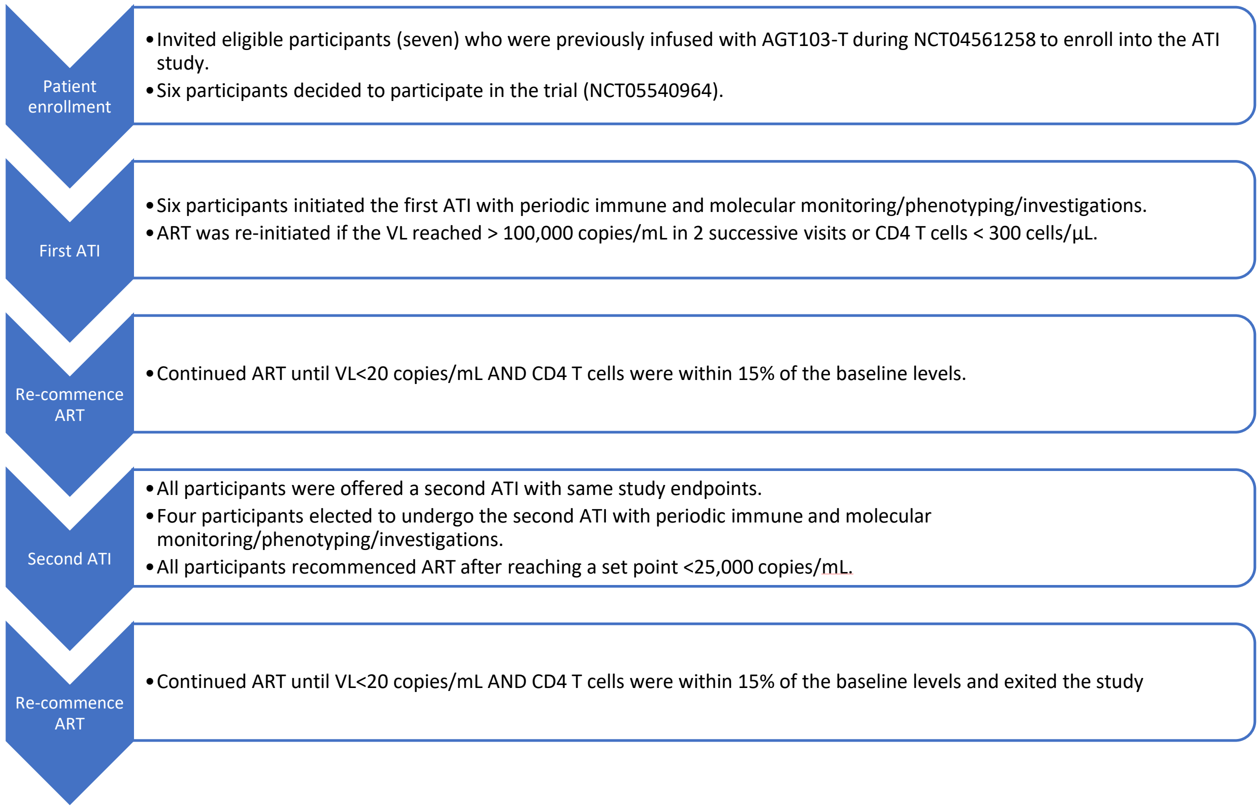

Figure S1: Clinical trial overview and study design.

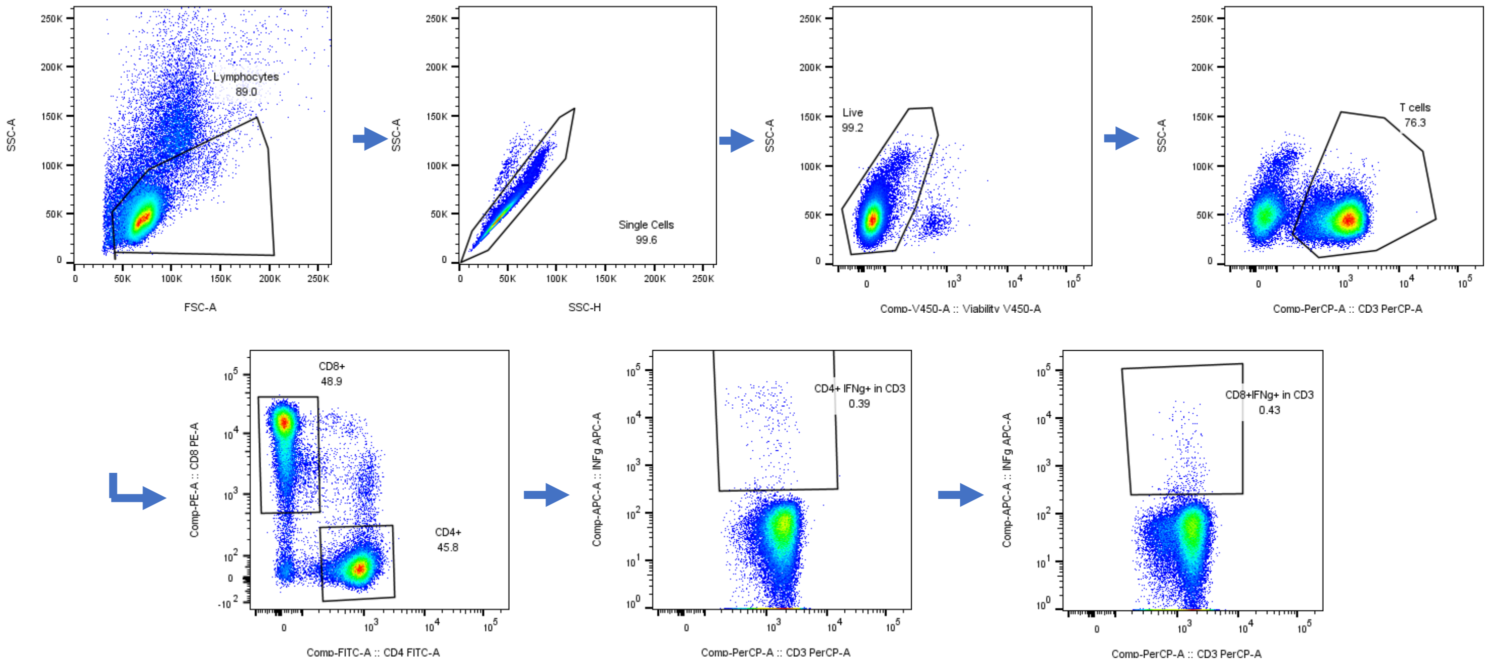

Figure S2: Gating strategy for ICS assay

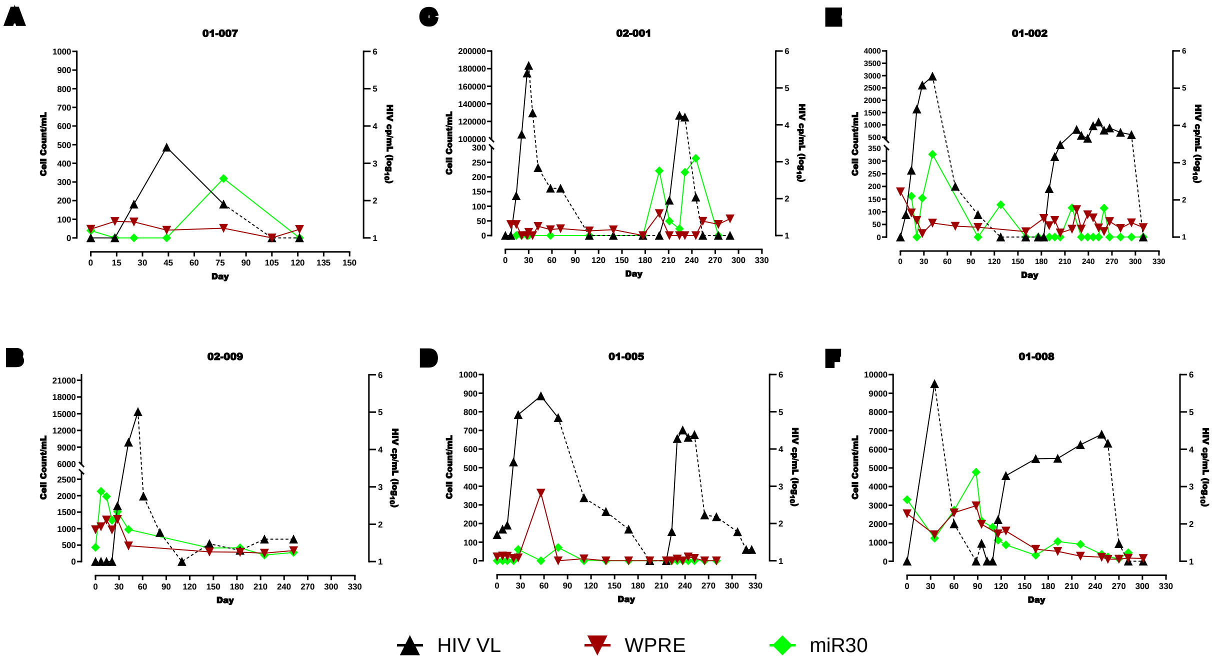

Figure S3: Comparison of AGT103-T cells detected by two independent methods. (A-F) AGT103-T cells detected at various timepoints during the study by two independent methods, qPCR and amplicon-sequencing in six study participants were plotted against the number of days (x-axis) and the viral load (black). The q-PCR method detected WPRE normalized against housekeeping RPL32 (red) and amplicon-seq detected the ratio of modified miR30 to native miR30 (green). The cell counts are plotted on the left-hand Y-axis while the VL is reported on the right-hand Y-axis. The solid black line indicates the VL during the ATI and the dashed black line represents the VL on ART.

Table S1: AGT103-T cells as a proportion of circulating CD4 T cells relative to the dose and duration between the infusion and the initiation of the ATI.

Patient ID	Infused Product Dose	Days between Infusion and the Start of ATI-1	AGT103-T (% of total CD4) at the Start of ATI-1
01-008	1.67 E+9	150	0.544
02-009	1.38 E+9	99	0.220
01-002	0.192 E+9	490	0.026
02-001	0.62 E+9	246	0.008
01-005	0.46 E+9	411	0.003
01-007	0.19 E+9	390	0.009
